# “Where’s Wally?” Identifying theory of mind in school-based social skills interventions

**DOI:** 10.3389/fpsyg.2022.600699

**Published:** 2022-10-26

**Authors:** Aneyn M. O’Grady, Sonali Nag

**Affiliations:** Department of Education, University of Oxford, Oxford, United Kingdom

**Keywords:** social skill, social cognition, intervention, configurative review, theory of mind (ToM), conceptual mapping, children, theoretical framework

## Abstract

This mini configurative review links theory of mind (ToM) research with school-based social skills interventions to reframe theoretical understanding of ToM ability based on a conceptual mapping exercise. The review’s aim was to bridge areas of psychology and education concerned with social cognition. Research questions included: how do dependent variables (DVs) in interventions designed to enhance child social-cognitive skills map onto ToM constructs empirically validated within psychology? In which ways do these mappings reframe conceptualization of ToM ability? Thirty-one studies (conducted from 2012 to 2019) on social-cognitive skill with typically-developing children ages 3–11 were included as opposed to explicit ToM trainings in light of an identified performance plateau on ToM tasks in children. Intervention DVs mapped onto the following ToM constructs in at least 87% of studies: “Representation of Others and/or Self,” “Knowledge/Awareness of Mental States,” “Attributions/Explanations of Mental States,” “Social Competence,” “Predicting Behavior,” and “Understanding Complex Social Situations.” The absence of false-belief understanding as an intervention DV indicated a lack of direct training in ToM ability. A hierarchy to further organize the review’s ToM framework constructs as either skills or competences within the construct of ‘Representation of Others and/or Self’ is proposed. Implications for the conceptualization of ToM and social-cognitive research as well as educational practice are discussed, namely how school social skill interventions conceptualize skill along a continuum in contrast to the common artificial dichotomous assessment of ToM skill (i.e., presence or lack), yet the development of ToM can nevertheless be supported by the school environment.

## Introduction

### Navigating the social-cognitive world of the developing child

“Where’s Wally?” (Handford, 1987) is a series of UK puzzle books wherein children must find Wally hidden amidst Escher-like crowds, often well-camouflaged amongst red-herring objects, matched to his iconic peppermint-striped shirt and bobble-hat. Finding Wally may not call for complex social-cognitive processing, but it does involve vaguely systematic pattern-seeking echoed in the purpose of this preliminary configurative review. Sifting through a myriad of constructs (often used interchangeably) to define and organize skills pertinent to a child’s ability to represent others’ mental states is not without difficulty. However, such theoretical reflection on the development of mentalizing ability instigated by Premack and Woodruff (1978) and further discussed by Wellman (2018) is a necessary step to adequately characterize a child’s understanding of mind and unite relevant research fields.

In 2012 Henry Wellman was awarded the G. Stanley Hall Award by the American Psychological Association for his contributions to developmental psychology, signaling an agreement across the field of the importance of further researching (a) the mechanisms of social development, and (b) how children come to acquire the sophisticated ability of ascribing mental states to others (i.e., theory of mind; ToM). Ten years on, it is of interest to consider how the field has evolved to define ToM within the larger context of research on social-cognitive development and within education. On a similar timescale since Wellman’s award, the past decade has seen an important increase in school-based programs to train social-cognitive skills as part of a curricular focus on social-emotional learning (SEL). The present study aims to reconsider the definition of ToM through a conceptual mapping exercise of social skill outcome variables in SEL intervention studies onto construct categories of ToM ability previously identified in the literature. This study is the first to the researchers’ knowledge to reframe the definition of ToM based on conceptual trends across the psychology and education research fields that have acted in parallel in their focus on developing social cognition.

Social cognition encompasses diverse abilities tied to navigating social situations such as attributing internal states, decoding social and emotional cues, and forming impressions (Fiske and Macrae, 2012; Carlston, 2013; Fiske, 2013; Verhaeghen and Hertzog, 2014). The construct of ToM has roots in philosophy, but ToM as a term in empirical research can be traced back to Premack and Woodruff (1978). ToM encompasses the understanding that others have thoughts and desires separate from our own, as well as the abilities to make inferences about others’ internal experiences, to predict and interpret their behavior. ToM as such can be said to serve as a foundation to social interaction and has been linked to various aspects of social cognition including self-regulation (Korucu et al., 2017), executive function (Qu et al., 2015; Duh et al., 2016; Jones et al., 2018; Wilson et al., 2018), acquiring greater insight into internal states of the self and others (Peterson et al., 2012; Hofmann et al., 2016), and interpersonal skills that support peer relationships and effective social interaction (Slaughter et al., 2015).

Considering ToM in relation to social cognition to further clarify its definition as a construct today is a task that calls for a complex and multi-layered conceptual framework. Byom and Mutlu (2013) provide a “working definition” of ToM by flagging three components synthesized from ToM measures including “shared world knowledge,” “perceiving social cues,” and “interpreting actions,” but do not provide further comment as to how to organize the ToM-related social-cognitive skills they identify across different research fields. The components proposed by Byom and Mutlu (2013) also raise the difficulty in defining ToM if constructs are treated at the same conceptual level, whether a ToM ability or a ToM cognition (Butterfill and Apperly, 2013), whether skill, competence, or domain.

Schaafsma et al. (2015) call for a “programmatic revision of ToM” (p. 67) that distinguishes psychological processes constituting ToM from associated concepts. Social neuroscience researchers have proposed a two-component model informed by the type of tasks used to assess ToM: the *social-cognitive*, wherein mental states are inferred *via* explicit verbal reasoning, and the *social-perceptual*, wherein mental states are inferred based on non-verbal cues (Tager-Flusberg and Sullivan, 2000; Meinhardt-Injac et al., 2018, 2020). Meinhardt-Injac et al. (2020) found that the social-cognitive ToM component aligned with general cognitive development in participants aged 11–25, whereas the social-perceptual component presented age-related performance shifts. However, the two-component model is less clear for defining ToM from a developmental perspective and when considering social-cognitive skill in an applied setting: the school environment.

### The present study

This study is the first to map outcome variables from school-based social-cognitive skill interventions to an *a priori* ToM framework to identify theoretical overlap across research fields concerned with social-cognitive development and reframe conceptualization of ToM ability. This approach can be described as a “framework synthesis framed by dimensions explicitly linked to particular perspectives” (Gough et al., 2012, p. 3). This study can be further characterized as a configurative review in its goal of synthesizing trends across a specific field and type of study to address research questions concerned with theoretical and methodological trends. The undertaken conceptual mapping exercise involved an inferential process that did not assume an intervention’s intent to enhance ToM. Instead, the review’s authors interpreted how school intervention outcome variables mapped onto ToM constructs. Conceptual mapping exercises have proved useful in the field of SEL (e.g., Wigelsworth et al., n.d.), most recently to identify core components of SEL by focusing on both pedagogical practice and the content of SEL intervention programs (Wigelsworth et al., 2022).

Theory of mind trainings do not usually adopt previously validated programs, opting for experimentally-developed protocols instead (e.g., Gola, 2012; Bianco et al., 2016; Lecce and Bianco, 2019). The intent of the current review was, however, to explore research on social-cognitive skill development in real-world settings and as such, did not focus on ToM trainings. The current review also did not focus on studies with populations that already had demonstrable or diagnosed adjustment problems, similar to Durlak et al.’s (2011) approach for their meta-analysis of school-based universal SEL interventions. The following research questions were addressed in the present review:

1. How do dependent variables chosen in school-based interventions designed to enhance child social-cognitive skills map onto ToM constructs empirically validated within psychology?
2. In which ways do these mappings reframe current conceptualization of ToM ability?

## Materials and methods

### Search methods used for review and article screening

Twelve databases in the fields of psychology, education, and linguistics were searched for social-cognitive skills interventions conducted from 2012 to March 2019. The search returned 10,458 records that were then uploaded to Rayyan QCRI, a systematic review web-based application. An initial pre-screening of titles and abstracts reduced the number of records to 141 based on the following inclusion criteria: (a) intervention is school-based, (b) study population includes typically developing children ages 3–11, and (c) full article is available and written in English. Screening of full texts for quality, the presence of a previously validated or CASEL-approved social skills program (Collaborative for Academic, Social, and Emotional Learning [CASEL], 2013; McLeod et al., 2017), and inclusion of social-cognitive skill DVs resulted in a final selection of 31 articles for this configurative review (*see* Table 1), marked with an asterisk in list of references. The first author and a research assistant read 20% (*n* = 27) of pre-screened articles, assigning a quality rating along a scale ranging from “1” (low quality) to “4,” (high quality), to establish inter-rater reliability. A Cohen’s kappa was calculated, *k* = 0.703 [95% CI (0.435, 0.971); *p* < 0.0005] that indicated moderate to good agreement (Altman, 1999; McHugh, 2012).

**TABLE 1:** Summary of interventions included in review grouped by study design including: Number of participants, child participant age band, social skills program selected for intervention, and intervention-targeted social-cognitive areas^a^.

Study [Country]	(n)^b^	Child age band (Years)/UK school year	Social skills program	Social-cognitive domain targeted by intervention program^c^				
				RS	RDM	S-A	S-M	SA
**2 × 2 Mixed model design**								
Ohl et al. (2013) [UK]	385	7 to 8/Year 3	Year 3 Pyramid Intervention	x		x	x	x
**Between-subjects design**								
Ashdown and Bernard (2012) [Australia]	103	4 to 7/Reception – Year 2	You Can Do It! Early Childhood Education Program (YCDI)	x	x	x	x	x
Graham et al. (2015) [USA]	64	8 to 11/Years 4 – 6	Best Foot Forward	x		x	x	x
Kourmousi et al. (2018) [Greece]	2439	7 to 9/Years 3 – 4	Steps for Life	x	x	x	x	x
**Cluster-randomized trial (CRT)**								
Crean and Johnson (2013) [USA]	779	8 to 11/Years 4 – 6	Providing Alternative Thinking Strategies (PATHS)	x		x	x	x
DiPerna et al. (2018) [USA]	700	6 to 7/Year 2	Social Skills Improvement System Classwide Intervention Program (SSIS-CIP)	x	x	x	x	x
**Interrupted time-series**								
Whitcomb and Merrell (2012) [USA]	88	6 to 7/Year 2	Strong Start	x		x	x	x
**Longitudinal study**								
Leadbeater et al. (2016) [Canada]	2477	6 to 10/Years 2 – 5	The WITS Program (Walk Away, Ignore, Talk it Out, Seek Help)	x	x		x	x
**Nested cohort**								
Myles-Pallister et al. (2014) [Australia]	683	8 to 10/Years 4 – 5	Aussie Optimism Positive Thinking Skills Program (AO-PTS)	x		x	x	x
**Sequential cohort-control**								
Fraser et al. (2014) [USA]	688	8 to 9/Year 4	Making Choices (MC) Program	x	x	x	x	x
**Quasi-experiment**								
Carvalho et al. (2017) [Portugal]	474	8 to 10/Years 4 – 5	MindUp	x		x	x	x
Coelho and Sousa (2017) [Portugal]	982	10 to 12/Year 6 – 7	Positive Attitude Program	x	x	x	x	x
El Hassan and Mouganie (2014) [Lebanon]	80	7 to 9/Years 2 – 4	Social Decision-Making Skills Curriculum (SDSC)	x	x	x	x	x
Finne and Svartdal (2017) [Norway]	399	11 to 14/Years 7 – 9	Social Perception Training	x	x	x	x	x
Gol-Guven (2017) [Turkey]	397	5:06 to 9:07/Years 2 – 5	Lions Quest Program: Skills for Growing	x	x	x	x	x
Goossens et al. (2012) [The Netherlands]	1223	5 to 11/Years 1, 4 and 6	Providing Alternative Thinking Strategies (PATHS)	x		x	x	x
Hoglund et al. (2012) [Canada]	432	6 to 7/Year 2	Walk away, Ignore it, Talk it out and Seek help (WITS)	x			x	x
Koposov et al. (2014) [Russia]	391	10 to 11/Year 6	Aggression Replacement Training (ART)	x	x	x	x	x
Pereira and Marques-Pinto (2017) [Portugal]	98	9 to 13/Years 6 – 9	Experiencing Emotions		x	x	x	x
Raimundo et al. (2013) [Portugal]	334	9 to 10/Year 5	Slowly but Steadily	x	x	x	x	x
Schonert-Reichl et al. (2012) [Canada]	613	8 to 12/Years 5 – 8	Roots of Empathy (ROE)	x		x	x	x
Wang and Goldberg (2017) [USA]	88	7 to 9/Year 4	Bullying Literature Project (BLP)-Moral Disengagement (MD)	x	x	x	x	x
Wu et al. (2016) [China]	214	8 to 10/Years 4 – 5	Let’s Be Friends (Adapted from the ‘Making Choices Program’)	x	x	x	x	x
**Randomized controlled trial (RCT)**								
Daunic et al. (2012) [USA]	1296	7 – 12/Years 3 – 7	Tools for Getting Along (TFGA)	x	x	x	x	x
DiPerna et al. (2016) [USA]	755	6 to 7/Year 2	Social Skills Improvement System-Classwide Intervention Program (SSIS-CIP)	x			x	x
Graves et al. (2017) [USA]	61	5 to 8/Years 1 – 3	Strong Start	x		x	x	x
Havighurst et al. (2015) [Australia]	408	4 to 9/Reception – Year 4	Child Component: materials drawn from ‘Exploring Together’ and ‘Fast Track child group’ School Component: Providing Alternative Thinking Strategies (PATHS) or Professional Learning Package (PLP)	x		x	x	x
Humphrey et al. (2016) [UK]	4516	7 to 9/Years 3 – 4	Providing Alternative Thinking Strategies (PATHS)	x		x	x	x
Low et al. (2015) [USA]	7400	5 to 8/Years 1 – 3	Second Step	x	x	x	x	x
Muratori et al. (2016) [Italy]	184	7 to 8/Year 3	Coping Power	x	x	x	x	x
Schonert-Reichl et al. (2015) [Canada]	99	9 to 11/Years 5 – 6	MindUP British Columbia (BC) Ministry of Education Social Responsibility Program	x	x	x	x	x

^a^ Based on the Collaborative for Academic, Social, and Emotional Learning (CASEL) framework to define SEL (Collaborative for Academic, Social, and Emotional Learning [CASEL], 2005).

^b^ The total number of participants at times includes teachers, school staff and parents who participated in the study in addition to child participants.

^c^ RS, relationship skills; RDM, responsible decision-making; S-A, self-awareness; S-M, self-management; SA: social awareness.

### Generating an *a priori* theory of mind framework to define child mind representation ability

Meta-analyses of studies conducted with children since 2012 were searched as well as seminal literature to select recent ToM framework constructs either explicitly cited or synthesized from study definitions of ToM. A brief report on a conceptual mapping exercise conducted for the Education Endowment Foundation SPECTRUM Database of non-academic skill assessment was also consulted (Wigelsworth et al., n.d.). The seven constructs to define ToM ability and respective sources from the research literature (meta-analyses are italicized) were as follows:^1^

1. Attributions/explanations of mental states (Wellman et al., 1996; Bosacki and Astington, 1999; Peterson et al., 2012; Hutchins et al., 2014; Wellman, 2014; Rakoczy, 2017; Wellman and Lind, 2020).
2. False-belief understanding (Wellman et al., 2001; Wellman and Liu, 2004; Shahaeian et al., 2011; Wellman, 2012, 2014; Hutchins et al., 2014; *Slaughter et al., 2015; Hofmann et al., 2016*; Wellman and Lind, 2020).
3. Knowledge/awareness of mental states (Flavell, 2004; Wellman and Liu, 2004; Hutchins et al., 2014; Tahiroglu et al., 2014; Wellman, 2014; *Slaughter et al., 2015; Hofmann et al., 2016*; Wellman and Lind, 2020).
4. Predicting behavior (Peterson et al., 2012; Hutchins et al., 2014; Wellman, 2014; *Slaughter et al., 2015*; Wellman and Lind, 2020).
5. Representation of others and/or self (Wellman et al., 1996; Carpendale and Lewis, 2004; Hutchins et al., 2014; Wellman, 2014; *Slaughter et al., 2015*; Wellman and Lind, 2020).
6. Social competence (Rose-Krasnor, 1997; Bosacki and Astington, 1999; Liddle and Nettle, 2006; Hughes, 2011; Peterson et al., 2012; Wellman, 2014; *Slaughter et al., 2015*; Wellman and Lind, 2020).
7. Understanding complex social situations (Baron-Cohen et al., 1999; Bosacki and Astington, 1999; Peterson et al., 2012; Hutchins et al., 2014; Wellman, 2014; *Hofmann et al., 2016*; Wellman and Lind, 2020).

## Conceptual mapping of social-cognitive skills intervention dependent variables to a seven-construct theory of mind framework

Dependent variables (DVs) were extracted from included articles, recorded verbatim, and then mapped onto the ToM framework. Conceptual mapping revealed that over 87% of outcome variables included in school-based social skill interventions mapped onto all ToM constructs included in the framework, except for false-belief understanding. ToM was not explicitly cited as a DV across analyzed interventions. Overall, recurrent DVs were abilities that could be extrapolated to involve ToM ability as they pertained to social problem-solving skills and the awareness of internal states. Supplementary Table 1 presents results of the mapping exercise with child-related outcome variables grouped thematically within each of the seven constructs of the ToM framework.

## Discussion

### Main trends from conceptual mapping exercise

School-based social skill intervention programs targeted at least three key social-emotional learning areas, namely “Relationship Skills,” “Self-Management,” and “Social Awareness.” To address the first research question, the conceptual mapping exercise revealed that intervention DVs mapped onto the following ToM constructs in at least 87% of studies: “Representation of Others and/or Self,” “Knowledge/Awareness of Mental States,” “Attributions/Explanations of Mental States,” “Social Competence,” “Predicting Behavior,” and “Understanding Complex Social Situations.” No school intervention DV mapped onto the “False-Belief Understanding” construct.

One emergent theme across DVs was a focus on child engagement with and potential behavioral impact on external referents, whether concerning behavior with peers and teachers (e.g., aggression) or within the classroom environment (e.g., on-task behavior). DVs focused on either reducing or promoting behaviors for social harmony, or self-cognition to then improve interpersonal relationships; both rely on the fundamental ability to represent others and the self (i.e., ToM). However, ToM was neither explicitly mentioned nor directly assessed across school social skill interventions despite their focus on representing others’ internal states and promoting social decision-making and/or problem-solving skill.

The absence of both ToM as a DV and of intervention DVs that mapped onto the ToM framework construct of false-belief understanding can be explained by theoretical and practical considerations. Although ToM is linked to social-cognitive skill, interventions included in this review focused on a broader scope of social-cognitive development rather than explicit ToM training. The predominant study age band (6–12 years) has also been identified as a performance plateau for traditional ToM tasks (Wellman, 2014) and consequently would have offered little insight for researchers assessing program impact. This potential age-based measure gap is of concern given that ToM continues to develop through middle childhood and adolescence (Wellman et al., 2011; Peterson and Wellman, 2018), developmental periods wherein individual differences in social cognition can persist into later social development (Dunn, 2000; Steinberg, 2005; Fuhrmann et al., 2015).

False-belief understanding is often used as a measure of ToM ability and as such, the lack of false-belief understanding as a DV is not surprising given the absence of ToM as an explicit outcome in included studies. Although a useful acquisition milestone for ToM development well-established in the literature (e.g., Happé et al., 2017), levels of false-belief understanding were not assessed in school-based intervention studies concerned with social-cognitive skill. As such, we encounter a limit to this review’s effort to unify research in psychology with that in education. One possible explanation is that school-based social-cognitive interventions are concerned with real-world interaction and train students using situations likely to be encountered. False-belief measures may be too specific and removed from this applied classroom setting to be relevant. Another explanation could be that school-based interventions conceptualize social-cognitive skill along a continuum, and often false-belief measures only allow for an “artificial” dichotomous assessment of skill (i.e., presence or lack) as raised by Liszkowski (2013, p. 105).

### Reframing theory of mind: Toward a comprehensive conceptual model

To answer the review’s second research question on how to reframe ToM ability, one main trend from the mapping exercise was the centrality of emotion awareness and regulation in social skill interventions. This was not surprising given that emotion understanding has been understood as a specific form of ToM (Ready et al., 2017), although the place of emotion as it relates to core ToM constructs is often subsumed within a broader ability to represent internal states. A category of *social emotions* that develops during puberty (Burnett et al., 2011) has been identified to characterize emotional states that incur the representation of others’ mental states (e.g., embarrassment, guilt). Such social emotions provide a theoretical bridge between emotion representation, social cognition more broadly, and ToM—often more concerned with the representation of non-affective states.

Furthermore, ToM has been linked to prosocial behavior (Caputi et al., 2012; Imuta et al., 2016), affective sensitivity (Contreras-Huerta et al., 2020) and perspective-taking (Tamnes et al., 2018) that all pertain to interpersonal interaction. The representation of an “other’s” mind can be said to be incomplete should emotional or affective states not be considered part of ToM ability. In effect, even while a theoretical difference is made within the literature between the ability to represent the thoughts, beliefs and desires of an “other” (i.e., cognitive ToM), and the ability to understand emotional states and preferences (i.e., affective ToM) (e.g., Sebastian et al., 2012), equal weight is not necessarily given in child development research. A nuanced framework for ToM ability could apprehend both cognitive and affective states within the broader construct of representing others and/or the self, in itself part of social cognition.

**FIGURE 1:**
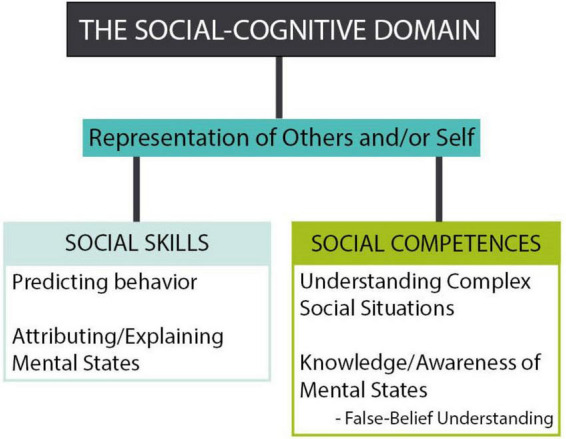
Proposed conceptual organization of the review’s core theory of mind (ToM) framework constructs based on mapping exercise of school-based social skills intervention outcomes onto the ToM framework. For reference, the seven constructs part of the original ToM framework used in the review’s mapping exercise included: (1) Attribution/explanation of mental states; (2) False-belief understanding; (3) Knowledge/awareness of mental states; (4) Predicting behavior; (5) Representation of others and/or self; (6) Social competence; (7) Understanding complex social situations. Constructs are listed in alphabetical order.

A further theoretical bridge between social skills and cognitive ability linked by ToM arises when considering another DV common to interventions analyzed here: social problem-solving skills. Social problem-solving skills emphasize the strategic component of ToM ability at times lost in research (Sher et al., 2014), as well as the adaptive and flexible thinking involved in how ToM is used in real-life social situations (Liszkowski, 2013). Given these trends, we propose the inductively identified ToM framework constructs be subsumed under one key framework construct—that of “Representation of Others and/or Self”—and organized as a skill or competence (*see* Figure 1). “Skill” is understood as a learned ability, “competence” as a repertoire of skills as compared to a level of performance ability in a given area, and “domain” as a field of mastery (American Psychological Association, 2022). We find the level of distinction between skill and competence necessary in the adapted framework as its absence is one source of confusion in organizing pertinent constructs for both ToM and social-cognitive development, as raised previously.

### Limitations of this study

The choice to look for ToM within a school setting while excluding ToM training studies may have skewed the search and findings from the conceptual mapping exercise in that it presents a specific analytic lens: a focus on universal social skill interventions. The review and mapping exercise present a novel effort to highlight how core constructs to define ToM within the psychological field are captured in social skill intervention outcomes, but do not speak to all efforts undertaken within schools to promote mentalizing ability. Inclusion criteria restricted interventions to a specific population, setting, and timeframe; this account of included interventions and choice of DV to assess social-cognitive skills is in no way exhaustive of all trends within this field. There was also an inherent difficulty during analysis to categorically bound ToM framework constructs, resulting in many mapping overlaps for the same DVs.

## Conclusion and future directions

Social-cognitive research acknowledges the complexity and multi-faceted nature of its empirical focus with a vast repertory of constructs, but there is an arguable lack of unity. Amongst the review’s 31 studies, there were no overwhelmingly recurrent DVs or common social skill programs. Construct operationalization greatly varied even in instances wherein the same DV was adopted. This review does not call for uniformity in developmental social-cognitive research, but rather attempts to synthesize in its aim of identifying shared theoretical trends based on a mapping exercise of social skills intervention outcomes to a ToM framework. Meaningful overlap between the majority of ToM constructs and school intervention outcomes was found, but a cohesive organization was strained by their variety and inter-relatedness. Nevertheless, the review proposes a novel organization of core ToM constructs (empirically established) to be grouped under a main construct of “Representation of Others/the Self” (in itself part of the social-cognitive domain), and further categorized as either skills or competences that we hope can prove useful for future research.

The lack of traditionally understood ToM and related milestone-construct of false-belief understanding as explicit DVs potentially points to an empirical shift in school-based interventions away from framing ToM as a skill that can be enhanced. However, the development of ToM understanding characterized as a “progression of conceptual achievements” (Wellman, 2018, p. 375) can be readily supported by learning within the school environment. Given ToM is tied to a majority of skills targeted by social-cognitive interventions, it does seem strange that ToM is not directly considered as enhancing mind-representing ability that would then logically impact on social-cognitive development. This is not necessarily problematic. Even within the context of developing ToM ability, researchers have advanced that direct training in mental-state concepts may not be the type of learning that best supports passing the ToM litmus test of the false-belief task, and that improved performance hinges on pragmatic language ability instead (Helming et al., 2014, 2016; Westra and Carruthers, 2017).

In fact, Wellman (2012) notes that much of ToM research is not necessarily developmental and calls for studies that capture a sequential progression of ability acquisition (e.g., microgenetic studies). Now in 2022 “social skill” is empty as an umbrella term to describe ToM, but the body of intervention research on social-cognitive development conducted since Wellman’s award has the potential to paint a cohesive picture of ToM as a set of skills and competences that can be systematically developed as seen here in the context of school-based programmes. Coupled with an effort to safeguard against jingle-jangle fallacies, further conceptual refinement of ToM will allow for convergence across social-cognitive research areas to consistently reflect ToM’s status as an ability crucial to development that can be enhanced for all children, as easy to spot as Wally’s red-and-white stripes.

## Author contributions

AO’G completed the initial search of databases for the review, screened articles for quality along with research assistants, extracted and analyzed the data, and drafted the main body of the manuscript. SN screened articles in cases of conflict between AO’G and research assistants, and provided feedback as well as edited the manuscript. Both the authors contributed to the article and approved the submitted version.

## Footnotes

1 Constructs are listed alphabetically.
