# Supplementary material for: “Where’s Wally?” Identifying theory of mind in school-based social skills interventions
Source: Front Psychol. 2022 Oct 26;13:600699. doi: 10.3389/fpsyg.2022.600699 (PMC9646489; doi:10.3389/fpsyg.2022.600699)
Supplement: Supplementary file 1 [file Table_1.docx]

| **Supplementary Table 1** | | |
| --- | --- | --- |
| *Conceptual Mapping of Dependent Variables Extracted from ‘n’ Social Skills Interventions onto a 7-Construct Theory of Mind (ToM) Framework** | | |
| **Representation of Others and/or Self** (*n* = 31) | | |
| **Emotional Intelligence**  (Interpersonal; Intrapersonal; Stress management; Adaptability; General Mood)  **(Child)** **Emotion Knowledge**  (emotion identification; emotion recognition in others; basic social problem-solving strategies; emotion understanding)  **Emotional Knowledge and Recognition**  (emotion attribution accuracy)  **Understanding of Infant Crying**  **Emotional Control**  (Cognitive Reappraisal; Expressive Suppression)  **Cognitive Control/Executive Function** (EF)    **Social-Emotional Development**  (Assertion; Cooperation; Self-Control)  **Social-Emotional Assets**  (coping skills; empathy; global self-concept; peer acceptance/relationships; problem-solving abilities; resilience/y in face of difficulties; social-emotional knowledge; use of coping and problem solving)  **Social-Emotional Competence(s)/ies**  (Academic Behavior; Assertion; Communication; Cooperation; Conduct Problems; Emotion Management; Emotional Knowledge—emotion perception accuracy in social behaviors, social situations, & facial expressions; Emotional Symptoms; Empathy; Engagement; Hyperactivity; Leadership; Peer Relations; Peer Relationship Problems; Problem Solving; Prosocial Behavior; Responsibility; Self-Control; Self-Management/Compliance; Sincerity; Skills for Learning; Social Awareness; Social Anxiety; Social Competence—Academic Behavior, Peer Relations, Self-Management/Compliance; Social Isolation) | **Social Decision-Making/Problem Solving Skill(s)**  (Child Readiness—Learning to Learn, self-control, social awareness; Social Decision Making Skill—Alternatives, Choose, Consequences, Feelings, Goals, Plan-and-check, Problems, Try-and-Rethink; Positive/Negative Problem Orientation; problem-solving style: Rational/Adaptive, Impulsive/Careless, Avoidance)  **Social Information-Processing Skills**  (1. Encoding, 2. Interpretation, 3. Goal formulation, 4. Response Choice)  **Child-Reported Help-Seeking Behavior**  **Using and Honoring Accounts**  (response to peer transgression—apology; denial; indifference; legitimate excuse)    **Hostile Attribution Bias**  **Aggressive Social Problem-Solving**  **Aggressive Interpersonal Negotiation Strategies**  **Cognitive Distortions**  (assuming the worst; blaming others; minimizing/labeling; self-serving)  **Social Skills**  (Assertion; Communication; Cooperation; Empathy; Engagement; Responsibility; Self-Control)  **Social Skills/Academic Motivation in the Context of Actual Behavior**  (Attributions of Peer Intent; Mastery Orientation; Motivational Strategies of Intermediate Risk-Taking)  **Social Skills and Problem Behavior**  (Assertion; Cooperation; Internalized/Externalized Behaviors; Responsibility; Self-Control) | **Parent-Reported Child Behavior**  (Oppositional Defiant Disorder; Conduct Disorder; Hyperactivity)  **Teacher Ratings of Behavior**  (Cooperation; Externalizing; Self-Control)  **Teacher-Reported Child Functioning** (Academic Skills; Emotional Regulation; Prosocial Behavior)  **Teacher-Reported Social-Emotional Adjustment**  (internalizing problems, physical aggression, social competence)  **Child Classroom/Student Behavior**  (bossiness; belittling; cooperation; helpfulness; leadership; peer acceptance/sociometric status; rudeness; selfishness; social competence; unresponsiveness)  **Classroom Performance**  (Learning Motivation; Math Skills; Prosocial Behavior; Reading Skills)  **Student Friendships**  **Personal/Social Skills in Classroom Environment**  (ability to control verbal/physical aggressiveness; ability to control victimization; ability to take responsibility; concentration of attention; emotion identification/expression; emotion management; empathy; friendship skills; participation/cooperation in class; problem-solving; use of spoken/written language)  **Student Conflict Resolution Skills**  **Student Perceptions of School**  (Academic Self-Esteem; Classroom Supportiveness; Liking School; Self-Esteem; Student Autonomy and Power of Affecting the Classroom Procedures; Trusting the Teacher)  **Learning Environment**  (Pupil perception of relationships between classmates, safety at school, teachers’ emotional support, and well-being) |
| **Representation of Others and/or Self** (*n* = 31) (*continued)* | | |
| **Social-Emotional Knowledge**  (emotion identification; emotion recognition in others; basic social problem-solving)  **Social-Emotional Skills**  (affective sharing of others’ emotions; Emotion Regulation/Deficits; Emotional Awareness; Interpersonal Relationships; peer social preference; Responsible Decision-Making—Interpersonal Negotiation, coordination of self/others when considering consequences of different actions; Relationship Skills—establish positive relationships, gain social acceptance; Self-Awareness—Perceiving/Understanding Emotion; Self-Management—Compliance with Rules/Expectations, Cooperation, Managing/Regulating Emotion, Self-Restraint; Social Awareness—Expressing/Labeling Emotion; Social Cooperation—following instructions from adults, cooperating/compromising with peers; Social Independence—within the domain of the peer group; Social Interaction—gaining/maintaining acceptance and friendship of others)  **Externalizing Behavior**  (Aggression; Attention; Conduct Problems)  **Externalizing/Internalizing Problems**  **Outside of School and at Home**  (Conduct Problems; Emotion Symptoms; Hyperactivity/Inattention; Peer Difficulties; Prosocial)  **Moral Disengagement**  (dehumanization; minimizing one’s agentic role; moral  justification)  **Bullying/Victimization**  (At School; Child Reported; Peer; Physical; Relational;  Verbal) | **Social Behavior and Affect**  (Peer Relations; Problem Behavior Internalizing/Externalizing)  **Child Behavior**  (Clinical, Adaptive, Educationally Related, Aggression)  **Aggression**  (proactive; reactive)  **Prosocial Behavior(s)**  **Prosociality**  (Empathy and Perspective-Taking; Peer Nominations; Social Responsibility)  **Prosocial and Aggressive/Antisocial Behaviors**  (cooperates/ive; empathic; fair; helps other kids when they have a problem; kind; shares; understands other kids’ point of view; Aggressive interpersonal behavior—physical/verbal; proactive, reactive and relational aggression; starts fights; breaks rules)  **Empathic Concern & Perspective-Taking**  **Problem Behaviors**  (Bullying; Depression Level; Internalizing—anxiety, depression; Externalizing—attention deficit and hyperactivity (ADH), oppositional defiant disorder (ODD), conduct problems, relational aggression; Hyperactive-Inattentive; Interference—distracts others, disrupts classroom functioning; Prosocial Behavior; Relational/Physical Victimization)  **Problem Behaviors and Social Behavior**  **Acting Out Behavioral Problems**  (disruption within classroom)  **Conduct Problems**  (socially deviant behavior in school) | **Self-Esteem**  (Academic—self- confidence, social attention, social attraction, student initiative, success/failure; Global Self—global self-worth)  **Self-Compassion**  (common humanity; isolation; mindfulness; over-identification; self-kindness; self-judgement)  **Attributions for Failure**  (a bad teacher; bad luck; lack of effort; low ability)  **Attitudes about Social Behavior and Academic Motivation**  (Social Behavior—legitimacy of aggression; Academic Motivation—academic self-concept; sense of control/mastery)  **Normative Beliefs about Aggression**  (General Beliefs—acceptability of verbal or physical aggression [proactive/reactive])  **Psychological Adjustment**  (Anxiety; Aggressiveness; Social Problems)  **Well-being**  (Depressive Symptoms; Emotional Control; Emotional Well-Being; Mindfulness; Optimism; Positive Self-Orientation; Positive Social Orientation; Positive Work Orientation; Psychological Well-Being; School Self-Concept—abilities, enjoyment and interest in school subjects; Social Well-Being)    **Mental Health Difficulties**  (Conduct Problems; Emotional Symptoms; Inattention/Hyperactivity; Peer Problems)  **Aggression and Emotional Problems**  (kicks, bites or hits other children; fights with other children; worries; cries easily; has headaches or stomachaches; appears unhappy or depressed)  **Anger Expression**  **Experience of Positive and Negative Affect**  **Mindfulness** |
| **Knowledge/Awareness of Mental States** (n = 29) | | |
| **(Child) Emotion Knowledge**  (emotion identification; emotion recognition in others; basic social problem-solving strategies; emotion understanding)  **Emotional Knowledge and Recognition**  (emotion attribution accuracy)  **Social-Emotional Knowledge**  (emotion identification; emotion recognition in others; basic social problem-solving)  **Hostile Attribution Bias**  **Emotional Intelligence**  (Interpersonal; Intrapersonal; Stress management; Adaptability; General Mood)  **Understanding of Infant Crying**  **Mindfulness**  **Emotional Control**  (Cognitive Reappraisal; Expressive Suppression)  **Experience of Positive and Negative Affect**  **Mental Health Difficulties**  (Emotional Symptoms; Peer Problems)  **Anger Expression**  **Aggression and Emotional Problems**  (fights with other children; worries; cries easily; appears unhappy or depressed)  **Externalizing/Internalizing Problems Outside of School and at Home**  (Emotion Symptoms; Peer Difficulties; Prosocial)  **Cognitive Control/Executive Function (EF)**  **Well-being**  (Emotional Control; Emotional Well-Being; Mindfulness; Optimism; Positive Self-Orientation; Positive Social Orientation; Psychological Well-Being)  **Psychological Adjustment**  (Anxiety, Aggressiveness, Social Problems) | **Social Skills**  (Assertion; Communication;  Cooperation; Empathy; Self-Control)  **Personal/Social Skills in Classroom Environment**  (ability to control verbal/physical aggressiveness; concentration of attention; emotion identification/expression; emotion management; empathy; friendship skills; problem-solving)  **Social-Emotional Development**  (Assertion; Cooperation; Self-Control)  **Social-Emotional Competence(s)/ies**  (Assertion; Communication; Cooperation; Emotion Management; Emotional Knowledge—emotion perception accuracy in social behaviors, social situations, & facial expressions; Empathy; Leadership; Peer Relations; Problem Solving; Prosocial Behavior; Self-Control; Self- Management/Compliance; Sincerity; Social Awareness; Social Competence—Peer Relations, Self-Management/Compliance)  **Social-Emotional Assets**  (coping skills; empathy; global self-concept; peer acceptance/relationships; problem-solving abilities; resilience/y in face of difficulties; social-emotional knowledge; use of coping and problem solving)  **Social Decision-Making/Problem Solving Skill(s)**  (Child Readiness—self-control, social awareness; Social Decision-Making Skill—Feelings; problem-solving style: Rational/Adaptive)  **Social Information-Processing Skills**  (1. Encoding, 2. Interpretation)  **Social Skills/Academic Motivation in the Context of Actual Behavior**  (Attributions of Peer Intent)  **Student Conflict Resolution Skills**  **Aggressive Social Problem-Solving**  **Aggressive Interpersonal Negotiation Strategies** | **Teacher Ratings of Behavior**  (Cooperation; Self -Control)  **Teacher-Reported Child Functioning**  (Emotional Regulation; Prosocial Behavior)  **Teacher-Reported Social-Emotional Adjustment**  (social competence)  **Social Behavior and Affect**  (Peer Relations)  **Child Behavior**  (Adaptive, Aggression)  **Externalizing Behavior**  (Aggression)  **Aggression**  (proactive; reactive)  **Child Classroom/Student Behavior**  (cooperation; helpfulness; leadership; social  competence)  **Classroom Performance**  (Prosocial Behavior)  **Student Friendships**  **Prosociality**  (Empathy and Perspective-Taking; Peer Nominations; Social Responsibility)  **Prosocial Behaviors**  (cooperates/ive; empathic; fair; helps other kids when they have a problem; kind; shares; understands other kids’ point of view)  **Prosocial and Aggressive/Antisocial**  **Behaviors**  (cooperates/ive; empathic; fair; helps other kids when they have a problem; kind; shares; understands other kids’ point of view; Aggressive interpersonal behavior—physical/verbal; proactive, reactive and relational aggression)  **Empathic Concern & Perspective-Taking** |
| **Knowledge/Awareness of Mental States** (n = 29) (*continued)* | | |
| **Social-Emotional Skills**  (affective sharing of others’ emotions; Emotion Regulation; Emotional Awareness; Interpersonal Relationships; Responsible Decision-Making—Interpersonal Negotiation, coordination of self/others when considering consequences of different actions; Relationship Skills—establish positive relationships, gain social acceptance; Self-Awareness—Perceiving/Understanding Emotion; Self-Management—Cooperation, Managing/Regulating Emotion, Self-Restraint; Social Awareness—Expressing/Labeling Emotion; Social Cooperation—cooperating/compromising with peers; Social Interaction—gaining/maintaining acceptance and friendship of others) | **Problem Behaviors**  (Depression Level; Internalizing—anxiety, depression; Externalizing relational aggression; Hyperactive-Inattentive; Prosocial Behavior)  **Problem Behaviors and Social Behavior** | **Attitudes about Social Behavior and Academic Motivation**  (Social Behavior—legitimacy of aggression)  **Normative Beliefs about Aggression**  (General Beliefs—acceptability of verbal or physical aggression [proactive/reactive])  **Cognitive Distortions**  (assuming the worst; blaming others; minimizing/labeling; self-serving)  **Moral Disengagement** (dehumanization; minimizing one’s agentic role; moral justification)  **Learning Environment**  (Pupil Perception of relationships between classmates, teachers’ emotional support, and well- being)  **Self-Esteem**  (Academic—self- confidence; social attention)  **Self-Compassion**  (mindfulness; over-identification; self-kindness; self-judgement) |
| **Attributions/Explanations of Mental States** (n = 29) | | |
| **(Child) Emotion Knowledge**  (emotion identification; emotion recognition in others; basic social problem-solving strategies; emotion understanding)  **Emotional Knowledge and Recognition**  (emotion attribution accuracy)  **Emotional Intelligence**  (Interpersonal; Intrapersonal; Stress management; Adaptability; General Mood)  **Understanding of Infant Crying**  **Social-Emotional Knowledge**  (emotion identification; emotion recognition in others; basic social problem-solving)  **Social-Emotional Skills**  (affective sharing of others’ emotions; Emotion Regulation/Deficits Emotional Awareness Interpersonal Relationships; Responsible Decision Making—Interpersonal Negotiation, coordination of self/others when considering consequences of different actions; | **Cognitive Distortions**  (assuming the worst; minimizing/labeling; self-serving)  **Social Skills/Academic Motivation in the Context of Actual Behavior**  (Attributions of Peer Intent)  **Hostile Attribution Bias**  **Moral Disengagement**  (dehumanization; minimizing one’s agentic role; moral justification)  **Empathic Concern & Perspective-Taking**  **Prosociality**  (Empathy and Perspective-Taking)  **Attitudes about Social Behavior & Academic Motivation**  (Social Behavior—legitimacy of aggression)  **Normative Beliefs about Aggression**  (General Beliefs—acceptability of verbal or physical aggression [proactive/reactive]) | **Social Decision-Making/Problem-Solving Skill(s)**  (Child Readiness—Learning to Learn, self-control, social awareness; Social Decision-Making Skill—Alternatives, Choose, Consequences, Feelings, Goals, Plan-and-check, Problems, Try-and-Rethink; Positive/Negative Problem Orientation; problem-solving style: Rational/Adaptive, Impulsive/Careless, Avoidance)  **Social Information-Processing Skills**  (1. Encoding, 2. Interpretation, 3. Goal formulation, 4. Response Choice)  **Student Conflict Resolution Skills**  **Aggressive Social Problem-Solving**  **Aggressive Interpersonal Negotiation Strategies**  **Mental Health Difficulties**  (Emotional Symptoms; Peer Problems)  **Psychological Adjustment**  (Anxiety; Aggressiveness; Social Problems) |
| **Attributions/Explanations of Mental States** (n = 29) (*continued*) | | |
| Relationship Skills—establish positive relationships; Self-Awareness—Perceiving/Understanding Emotion; Self-Management—Cooperation, Managing/Regulating Emotion, Social Awareness—Expressing/Labeling Emotion; Social Cooperation—cooperating/compromising with peers)  **Social Skills**  (Communication; Cooperation; Empathy; Self-Control)  **Personal/Social Skills in Classroom Environment**  (ability to take responsibility; emotion identification/expression; emotion management; empathy; friendship skills; problem-solving; use of spoken/written language)  **Self-Esteem**  (Academic—social attention)  **Social-Emotional Assets**  (coping skills; empathy; peer acceptance/relationships; problem-solving abilities; resilience/y in face of difficulties; social-emotional knowledge; use of coping and problem-solving)  **Social-Emotional Competence(s)/ies**  (Communication; Cooperation; Emotion Management; Emotional Knowledge—emotion perception accuracy in social behaviors, social situations, & facial expressions; Emotional Symptoms; Empathy; Peer Relations; Peer Relationship Problems; Problem Solving; Prosocial Behavior; Self-Control; Self-Management; Social Awareness; Social Anxiety; Social Competence—Peer Relations, Self-Management) | **Using and Honoring Accounts**  (response to peer transgression)  **Learning Environment**  (Pupil Perception of relationships between classmates, teachers’ emotional support, and well-being)  **Child Classroom/Student Behavior**  (cooperation; helpfulness; leadership; social competence)  **Classroom Performance**  (Prosocial Behavior)  **Teacher Ratings of Behavior**  (Cooperation; Externalizing; Self-Control)  **Social-Emotional Development**  (Cooperation; Self-Control)  **Prosocial Behavior(s)**  **Prosocial and Aggressive/Antisocial Behaviors**  (cooperates/ive; empathic; fair; helps other kids when they have a problem; kind; understands other kids’ point of view; Aggressive interpersonal behavior—reactive and relational aggression; starts fights)  **Problem Behaviors**  (Depression Level; Internalizing—anxiety, depression; Externalizing—relational aggression; Prosocial Behavior; Relational/Physical Victimization)  **Social Behavior and Affect**  (Peer Relations; Problem Behavior: Internalizing/Externalizing)  **Student Friendships** | **Teacher-Reported Child Functioning**  (Emotional Regulation; Prosocial Behavior)  **Teacher-Reported Social-Emotional Adjustment**  (internalizing problems, social competence)  **Well-being**  (Depressive Symptoms; Emotional Control; Emotional Well-Being; Mindfulness; Optimism; Psychological Well-Being)  **Emotional Control**  (Cognitive Reappraisal; Expressive Suppression)  **Anger Expression**  **Experience of Positive and Negative Affect**  **Cognitive Control/Executive Function (EF)**  **Mindfulness**  **Self-Compassion**  (mindfulness; over-identification; self-kindness; self-judgement)  **Child Behavior**  (Adaptive, Aggression)    **Aggression**  (reactive)  **Aggression and Emotional Problems**  (fights with other children; worries; cries easily)  **Externalizing Behavior**  (Aggression)  **Externalizing/Internalizing Problems Outside of School and at Home**  (Emotion Symptoms; Peer Difficulties; Prosocial) |
| **Social Competence** (n = 28) | | |
| **Social Behavior and Affect**  (Peer Relations)  **Social Decision-Making/Problem- Solving Skill(s)**  (Child Readiness—Learning to Learn, self-control, social awareness; Social Decision-Making Skill—Alternatives, Choose, Consequences, Feelings, Goals, Plan-and-check, Try-and-Rethink; Positive/Negative Problem Orientation; problem-solving style—Rational/Adaptive)  **Child-Reported Help-Seeking Behavior**  **Social Information-Processing Skills**   1. Encoding, 2. Interpretation, 3. Goal formulation, 4. Response Choice)   **Student Conflict Resolution**  **Using and Honoring Accounts**  (response to peer transgression)  **Child Behavior**  (Adaptive)  **Child Classroom/Student Behavior**  (cooperation; helpfulness; leadership; peer acceptance/sociometric status; social competence)  **Classroom Performance**  (Prosocial Behavior)  **Teacher Ratings of Behavior**  (Cooperation; Self-Control)  **Teacher-Reported Child Functioning**  (Emotional Regulation; Prosocial Behavior)  **Teacher-Reported Social-Emotional Adjustment**  (social competence)  **Personal/Social Skills in Classroom Environment**  (ability to control verbal/physical aggressiveness; ability to control victimization; ability to take responsibility; emotion identification/expression; emotion management; empathy; friendship skills; problem-solving; use of spoken/written language)  **Student Friendships**  **Prosocial Behavior(s)**  **Prosociality**  (Empathy and Perspective-Taking; Peer Nominations; Social Responsibility) | **Attitudes about Social Behavior and Academic Motivation**  (Social Behavior—legitimacy of aggression)  **Normative Beliefs about Aggression**  (General Beliefs—acceptability of verbal or physical aggression [proactive/reactive])  **Learning Environment**  (Pupil Perception of relationships between classmates and teachers’ emotional support)  **(Child) Emotion Knowledge**  (emotion recognition in others; basic social problem-solving strategies; emotion understanding)  **Emotional Knowledge and Recognition**  (emotion attribution accuracy)  **Mindfulness**  **Understanding of Infant Crying**  **Emotional Intelligence**  (Interpersonal; Intrapersonal; Stress management; Adaptability; General Mood)  **Emotional Control**  (Cognitive Reappraisal; Expressive Suppression)  **Anger Expression**  **Cognitive Control/Executive Function (EF)**  **Self-Esteem**  (Academic—self-confidence, social attention, social attraction, student initiative, success/failure; Global Self—global self-worth)  **Self-Compassion**  (common humanity; mindfulness; self-kindness)  **Psychological Adjustment**  **Well-being**  (Emotional Control; Emotional Well-Being; Mindfulness; Optimism; Positive Self-Orientation; Positive Social Orientation; Psychological Well-Being; Social Well-Being)  **Using and Honoring Accounts**  (response to peer transgression—apology; denial; indifference; legitimate excuse) | **Social-Emotional Competence(s)/ies**  (Assertion; Communication; Cooperation; Emotion Management; Emotional Knowledge—emotion perception accuracy in social behaviors, social situations, & facial expressions; Empathy; Leadership; Peer Relations; Problem Solving; Prosocial Behavior; Responsibility; Self-Control; Self- Management/Compliance; Sincerity; Social Awareness; Social Competence—Peer Relations, Self-Management/Compliance)  **Social-Emotional Development**  (Assertion; Cooperation; Self-Control)  **Social-Emotional Knowledge**  (emotion identification; emotion recognition in others; basic social problem-solving)  **Social Skills**  (Assertion; Communication; Cooperation; Empathy; Engagement; Responsibility; Self-Control)  **Social Skills/Academic Motivation in the Context of Actual Behavior**  (Attributions of Peer Intent)  **Social-Emotional Skills**  (affective sharing of others’ emotions; Emotion Regulation; Emotional Awareness; Interpersonal Relationships; peer social preference; Responsible Decision-Making—Interpersonal Negotiation, coordination of self/others when considering consequences of different actions; Relationship Skills—establish positive relationships, gain social acceptance; Self-Awareness—Perceiving/Understanding Emotion; Self-Management—Compliance with Rules/Expectations, Cooperation, Managing/Regulating Emotion, Self-Restraint; Social Awareness—Expressing/Labeling Emotion; Social Cooperation—following instructions from adults, cooperating/compromising with peers; Social Independence—within the domain of the peer group; Social Interaction—gaining/maintaining acceptance and friendship of others) |
| **Social Competence** (n = 28) (*continued*) | | |
| **Prosocial Behaviors**  (cooperates/ive; empathic; fair; helps other kids when they have a problem; kind; shares; understands other kids’ point of view)  **Empathic Concern & Perspective-Taking** |  | **Social-Emotional Assets**  (coping skills; empathy; global self-concept; peer acceptance/relationships; problem-solving abilities; resilience/y in face of difficulties; social-emotional knowledge; use of coping and problem-solving) |
| **Predicting Behavior** (n = 27) | | |
| **Emotional Intelligence**  (Interpersonal; Intrapersonal; Stress management; Adaptability; General Mood)  **(Child) Emotion Knowledge**  (emotion identification; emotion recognition in others; basic social problem-solving strategies; emotion understanding)  **Emotional Knowledge and Recognition**  (emotion attribution accuracy)  **Understanding of Infant Crying**  **Social-Emotional Knowledge**  (emotion identification; emotion recognition in others; basic social problem-solving)  **Mindfulness**  **Social-Emotional Assets**  (empathy; peer acceptance/relationships; problem-solving abilities; social-emotional knowledge; use of coping and problem-solving)  **Social-Emotional Competence(s)/ies**  (Cooperation; Emotional Knowledge—emotion perception accuracy in social behaviors, social situations, & facial expressions; Empathy; Leadership; Peer Relations; Peer Relationship Problems; Problem Solving; Prosocial Behavior; Social Awareness; Social Competence—Peer Relations)  **Teacher-Reported Social-Emotional Adjustment**  (social competence)  **Prosociality**  (Empathy and Perspective-Taking)  **Empathic Concern & Perspective-Taking** | **Social Skills**  (Cooperation; Empathy)  **Personal/Social Skills in Classroom Environment**  (emotion identification; empathy; friendship skills; cooperation in class; problem-solving)  **Social-Emotional Skills**  (affective sharing of others’ emotions; Emotional Awareness; Interpersonal Relationships; Responsible Decision-Making—Interpersonal Negotiation, coordination of self/others when considering consequences of different actions; Relationship Skills—establish positive relationships, gain social acceptance; Social Awareness—Expressing/Labeling Emotion; Social Cooperation—cooperating/compromising with peers; Social Interaction—maintaining acceptance and friendship of others)  **Prosocial Behavior(s)**  (cooperates/ive; empathic; helps other kids when they have a problem; understands other kids’ point of view)  **Classroom Performance**  (Prosocial Behavior)  **Child Classroom/Student Behavior**  (cooperation; helpfulness; leadership; social competence)  **Teacher Ratings of Behavior**  (Cooperation)  **Teacher-Reported Child Functioning**  (Prosocial Behavior) | **Child Behavior**  (Adaptive)  **Problem Behaviors and Social Behavior**  **Social Behavior and Affect**  (Peer Relations)  **Psychological Adjustment**  (Social Problems)  **Mental Health Difficulties**  (Peer Problems)  **Student Friendships**  **Social Decision-Making/Problem- Solving Skill(s)**  (Child Readiness—social awareness; Social Decision-Making Skill—Alternatives, Choose, Consequences, Feelings, Goals, Plan-and-check, Try-and-Rethink)  **Student Conflict Resolution Skills**  **Child-Reported Help-Seeking Behavior**  **Aggressive Social Problem-Solving Aggressive Interpersonal Negotiation Strategies**  **Cognitive Control/Executive Function (EF)**  **Social Information-Processing Skills**   1. Encoding, 2. Interpretation, 3. Goal formulation, 4. Response Choice)   **Normative Beliefs about Aggression**  (General Beliefs—acceptability of verbal or physical aggression [proactive/reactive])  **Hostile Attribution Bias** |
| **Understanding Complex Social Situations** (n = 27) | | |
| **Emotional Intelligence**  (Interpersonal; Intrapersonal; Stress management; Adaptability; General Mood)  **(Child) Emotion Knowledge**  (emotion identification; emotion recognition in others; basic social problem-solving strategies; emotion understanding)  **Emotional Knowledge and Recognition**  (emotion attribution accuracy)  **Social-Emotional Knowledge**  (emotion identification; emotion recognition in others; basic social problem-solving)  **Mindfulness**  **Attitudes about Social Behavior and Academic Motivation**  (Social Behavior—legitimacy of aggression)  **Normative Beliefs about Aggression**  (General Beliefs—acceptability of verbal or physical aggression [proactive/reactive])  **Moral Disengagement**  (moral justification)  **Cognitive Distortions**  (assuming the worst; blaming others; minimizing/labelling)  **Social-Emotional Assets**  (empathy; peer relationships; problem-solving abilities; social-emotional knowledge; use of coping and problem-solving)  **Social-Emotional Competence(s)/ies**  (Cooperation; Emotional Knowledge—emotion perception accuracy in social behaviors, social situations, & facial expressions; Empathy; Leadership; Peer Relations; Peer Relationship Problems; Problem Solving; Prosocial Behavior; Responsibility; Social Awareness; Social Competence—Peer Relations) | **Personal/Social Skills in Classroom Environment**  (emotion identification; empathy; friendship skills; problem-solving)  **Social Skills**  (Empathy)  **Social Skills/Academic Motivation in the Context of Actual Behavior**  (Attributions of Peer Intent)  **Social-Emotional Skills**  (Emotional Awareness; Interpersonal Relationships; Responsible Decision-Making—Interpersonal Negotiation, coordination of self/others when considering consequences of different actions; Relationship Skills—establish positive relationships, gain social acceptance; Self-Awareness—Perceiving/Understanding Emotion; Self-Management—Cooperation; Social Awareness—Expressing/Labeling Emotion; Social Cooperation—cooperating/compromising with peers; Social Interaction—gaining/maintaining acceptance and friendship of others)  **Empathic Concern and Perspective-Taking**  **Social-Emotional Development**  (Cooperation)  **Psychological Adjustment**  (Social Problems)  **Teacher-Reported Social-Emotional Adjustment**  (social competence)  **Child Behavior**  (Adaptive)  **Social Behavior and Affect**  (Peer Relations)  **Teacher Ratings of Behavior**  (Cooperation)  **Teacher-Reported Child Functioning**  (Prosocial Behavior)  **Student Friendships** | **Cognitive Control/Executive Function (EF)**  **Social Decision-Making/Problem- Solving Skill(s)**  (Child Readiness—social awareness; Social Decision-Making Skill—Alternatives, Choose, Consequences, Feelings, Goals, Plan-and-check, Problems, Try-and-Rethink; Positive/Negative Problem Orientation)  **Social Information-Processing Skills**   1. Encoding, 2. Interpretation, 3. Goal formulation, 4. Response Choice)   **Using and Honoring Accounts**  (response to peer transgression—apology; denial; indifference; legitimate excuse)  **Student Conflict Resolution Skills**  **Child-Reported Help-Seeking Behavior**  **Aggressive Social Problem-Solving Aggressive Interpersonal Negotiation Strategies** |
| **False-Belief Understanding** (n = 0) | | |
| *Stated DVs across studies are aligned to the left and bolded; words in parentheses reflect measure items/sub-scale labels to contextualize DV operationalization. If DVs were similar in target construct, but used slightly different terms across studies (e.g., ‘Social-Emotional Competence’ vs. ‘Social-Emotional Competences’ vs. ‘Social-Competencies’), these cases are reflected by the use of a slash. | | |
